# Two Novel Homozygous *HPS6* Mutations (Double Mutant) Identified by Whole-Exome Sequencing in a Saudi Consanguineous Family Suspected for Oculocutaneous Albinism

**DOI:** 10.3390/life12010014

**Published:** 2021-12-23

**Authors:** Sajjad Karim, Samah Saharti, Nofe Alganmi, Zeenat Mirza, Ahmed Alfares, Shereen Turkistany, Manal Al-Attas, Hend Noureldin, Khadega Al Sakkaf, Heba Abusamra, Mohammed Al-Qahtani, Adel Abuzenadah

**Affiliations:** 1 Center of Excellence in Genomic Medicine Research, Faculty of Applied Medical Sciences, King Abdulaziz University, Jeddah 21589, Saudi Arabia; mhalqahtani@kau.edu.sa (M.A.-Q.); aabuzenadah@kau.edu.sa (A.A.); 2 Department of Medical Lab Technology, Faculty of Applied Medical Sciences, King Abdulaziz University, Jeddah 21589, Saudi Arabia; zmirza1@kau.edu.sa; 3 Department of Pathology, Faculty of Medicine, King Abdulaziz University, Jeddah 21589, Saudi Arabia; snsaharti@kau.edu.sa; 4 Computer Science Department, Faculty of Computing and Information Technology, King Abdulaziz University, Jeddah 21589, Saudi Arabia; nalghanimi@kau.edu.sa; 5 King Fahd Medical Research Center, Faculty of Applied Medical Sciences, King Abdulaziz University, Jeddah 21589, Saudi Arabia; 6 Division of Genetics, Department of Pediatrics, King Abdulaziz Medical City, Riyadh 11426, Saudi Arabia; fars@qu.edu.sa; 7 Center of Innovation in Personalized Medicine, King Abdulaziz University, Jeddah 21589, Saudi Arabia; sturkistany@kau.edu.sa; 8 Roya Specialized Medical Laboratories, King Abdulaziz University, Jeddah 21589, Saudi Arabia; mhhalattas@kau.edu.sa (M.A.-A.); hnoureldin@kau.edu.sa (H.N.); khsaggaf14@gmail.com (K.A.S.); heba.abusamra@kaust.edu.sa (H.A.)

**Keywords:** oculocutaneous albinism, Hermansky-Pudlak syndrome 6, whole-exome sequencing, *HPS6* gene, double mutation, Saudi Arabia

## Abstract

Background: Oculocutaneous albinism (OCA) is an autosomal recessive disorder of low or missing pigmentation in the eyes, hair, and skin. Multiple types of OCA, including Hermansky-Pudlak syndrome 6 (*HPS6*), are distinguished by their genetic cause and pigmentation pattern. *HPS6* is characterized by OCA, nose bleeding due to platelet dysfunction, and lysosome storage defect. To date, 25 disease-associated mutations have been reported in the *HPS6* gene. Methods: DNA was extracted from proband, and whole-exome sequencing (WES) was performed using the Illumina NovaSeq platform. Bioinformatic analysis was done with a custom-designed filter pipeline to detect the causative variant. We did Sanger sequencing to confirm the candidate variant and segregation analysis, and protein-based structural analysis to evaluate the functional impact of variants. Result: Proband-based WES identified two novel homozygous mutations in *HPS6* (double mutation, c.1136C>A and c.1789delG) in an OCA suspect. Sanger sequencing confirmed the WES results. Although no platelet and/or lysosome storage defect was detected in the patient or family, an oculocutaneous albinism diagnosis was established based on the *HPS6* mutations. Structural analysis revealed the transformation of abnormalities at protein level for both nonsense and frameshift mutations in *HPS6*. Conclusion: To the best of our knowledge, the double mutation in *HPS6* (p.Ser379Ter and p.Ala597GlnfsTer16) represents novel pathogenic variants, not described previously, which we report for the first time in the Saudi family. In silico analyses showed a significant impact on protein structure. WES should be used to identify *HPS6* and/or other disease-associated genetic variants in Saudi Arabia, particularly in consanguineous families.

## 1. Introduction

Oculocutaneous albinism (OCA) is characterized by reduced or lack of melanin pigment in the skin, hair, and eyes. These conditions are brought about by transformations in explicit qualities that are important for creating melanin shade in particular melanocytes. Missing or deficiency of melanin may cause vision anomalies and fair but defenseless skin to be harmed from the sun, leading to skin malignancy [1,2]. Vision issues associated with OCA include abnormal eyes movement (nystagmus), diminished iris color, decreased retinal shade, macular hypoplasia, poor foveal/central visual sharpness, and poor nerves to cerebrum association (strabismus) [3].

Multiple types of oculocutaneous albinism, including type I to VIII, have been recognized by their pigmentation pattern and genetic reason. Mutations in *TYR, OCA2, OCA5, TYRP1, SLC45A2/MATP, SLC24A5, LRMDA,* and *TYRP2* genes are known to cause OCA type 1 to type VIII, respectively [4,5,6,7,8,9,10,11,12]. Additionally, mutations in the *HPS, CHS/LYST, MC1R,* and *OA1* genes have also been reported to cause different types of OCA through Hermansky-Pudlak syndrome (MIM# 203300), red-haired OCA2 (MIM#203200), Chediak–Higashi syndrome (MIM# 214500), and X-linked ocular albinism (MIM#300500), respectively [12,13,14,15,16].

Hermansky-Pudlak syndrome (HPS) is an autosomal recessive disorder with worldwide incidence of 1 in 500,000–1,000,000 featuring OCA, visual disability, bleeding diathesis, and melanosomes/platelet granules and lysosomal storage defects. In some affected individuals, stockpiling of lysosomal ceroid lipofuscin, colitis, lung fibrosis, or immunodeficiency is also reported [14,17,18]. Ocular issues include reduced iris and retinal pigment, reduced visual acuity, and nystagmus, while color shades range from white to brown for hair, and white to olive for skin [19].

To date, mutations in *HPS1* (MIM#604982,10q24), *AP3B1*(MIM#603401, 5q14), *HSP3* (MIM#606118, 3q24), *HSP4* (MIM#606682,22q12), *HPS5* (MIM#607521, 11p14), *HPS6* (MIM#607522, 10q24), *DTNBP1* (MIM#607145, 6p22), *BLOC1S3* (MIM#609762,19q13), *PLDN* (MIM# 604310, 15q21), *AP3D1* (MIM#607246, 19p13), and *BLOC1S5* (MIM#607289, 6p24) genes have been reported to cause 11 types of genetic heterogeneity in HPS from HPS1 to HPS11, respectively [18,20,21,22,23]. The *HPS6* gene has a single large exon mapped at chromosome 10q24.32, and expressed proteins are involved in pigment biogenesis through lysosome and related organelles, and mutations in this gene cause HPS type 6 [20,23].

A patient with lack of eye pigment, visual impairment, and nystagmus was suspected as OCA. Because of genetic heterogeneity of OCA and no established systematic genetic analysis being followed for albinism in high consanguineous/endogamous families of the Arabian Peninsula, it was challenging to scan all potential genetic variants by direct Sanger sequencing. Whole-exome sequencing (WES) has been used frequently for the diagnosis of genetic disorders, including OCA [17,24]. We, therefore, carried out WES to identify novel deleterious mutations in syndromic OCA-associated genes as the human exome covers ~85% of known disease-related variants [25]. Further, the segregation pattern of this variant in the family was confirmed by Sanger and the impact of the variant was evaluated by structural analysis.

## 2. Materials and Methods

### 2.1. Patient and Ethical Approval

A proband (6-year-old male) had symptoms of visual impairment and lack of pigmentation of skin and hair; the referring clinician at King Abdulaziz University Hospital suspected OCA, a rare inherited disorder of melanin biosynthesis. He had a positive family history; thus, we recruited this Saudi family and based on interviewing the family, a pedigree was constructed carefully. Written consent was obtained from the patient’s parents. This study was approved by the local ethical committee of King Abdulaziz University (01-CEGMR-Bioeth-2021) and was conducted according to the principles of the Declaration of Helsinki.

### 2.2. DNA Isolation

DNA was extracted from peripheral blood samples of the affected family using the manufacturer’s protocol using QIAamp genomic DNA extraction kit (QIAGEN, Germantown, MD, USA). DNA quantification and quality assessment was done by NanoDrop spectrophotometer (Thermo Fisher Scientific, Waltham, MA, USA) and agarose gel electrophoresis was done to confirm the integrity of genomic DNA before sequencing [26,27].

### 2.3. Whole-Exome Sequencing

Whole-exome sequencing (WES) was performed by loading 100 μL of quantified DNA (70–80 ng/μL) on the flow cell of NovaSeq™ 600 Sequencing System (Illumina, San Diego, CA, USA). The Nextera™ DNA Flex Pre-Enrichment Library Prep and whole exome-enrichment kit (Illumina, San Diego, CA, USA) were used for library formulation and the exome enrichment of exonic and intron flanking regions based on the manufacturer protocol. DNA fragmentation, tagmentation, purification, amplification, target capturing, and enrichment were conducted using magnetic beads, Qubit fluorometer, and Illumina reagent kits as per the manufacturer’s protocol, respectively [28,29,30].

### 2.4. Exome Sequencing Data Analysis

The base call reads (BCL) of WES output were converted to raw reads (FASTQ) using BCL2FASTQ software. Quality assessment and pre-aligned processing was done using FastQC and Trimmonatic. The reads were aligned with reference sequences (human genome build GRCh37/UCSChg19) to generate a binary aligned map (BAM) file using the BWA mem algorithm and SAMtool. After removing read duplicates (Picard), the base quality score was calibrated (GATK-BaseRecalibrator ReQON), and variants calls (VCF) were selected, annotated, and analyzed using ANNOVAR and the Genome Analysis Tool Kit (GATK HaplotypeCaller, http://www.broadinstitute.org/gatk, accessed on 1 November 2021) [28,30,31].

### 2.5. Variant Filtration and Prioritization

To identify the disease causative variants, the BaseSpace Variant Interpreter (Illumina, USA) was used for variant filtration and prioritization. The Human Genome Variation Society (HGVS) nomenclature guidelines were used to describe variants. WES data were analyzed and filtered to identify causative pathogenic variants based on base quality (Phred score > 30), rare population allele frequency (MAF < 0.01), genomic position (coding and splice site), impact (amino acid change and premature termination of protein), pathogenicity (SIFT, Polyphen, and CADD), allelic zygosity (homozygous or heterozygous in patients), and association with the disease phenotype (ClinVar, VarSome). Minor allele frequency ≤ 0.01 was applied to filter out common variants in population using open databases like the gnomAD—Genome Aggregation Database (https://gnomad.broadinstitute.org/, accessed on 28 October 2021), ExAC—Exome Aggregation Consortium, EVS—Exome Variant Server (https://evs.gs.washington.edu/EVS/, accessed on 27 October 2021), the TopMed—TransOmics for Precision Medicine (https://topmed.nhlbi.nih.gov/, accessed on 25 October 2021), SGHP—Saudi Human Genome Project, and the 1000 Genomes Consortium (https://www.internationalgenome.org/, accessed on 30 October 2021). Variants were considered novel or rare for their absence or low allele frequency in the reported databases. We included coding consequences, such as stop gain or loss, splice site, frameshift, indels, and missense mutations with most severe impacts, and we excluded CNVs and SVs. Data analysis using gene-specific filtering and a literature/database search was performed to limit the variants/genes relevant to the patients’ clinical history. We searched for predicted pathogenic and/or likely pathogenic variants and any of the previously reported ClinVar variants. Pathogenicity of variants were classified as per the American College of Medical Genetics and Genomics (ACMGG) guidelines [32]. Family history and pedigree were used to hypothesize the zygosity and mode of inheritance: autosomal recessive (AR), autosomal dominant (AD), or X-linked (XL). Additionally, the predicted VUS (damaging in SIFT/Polyphen) associated with patients’ phenotypes was searched. Any null variant (nonsense, frameshift) with a reported loss-of-function (LOF) mechanism for causing a disease (PVS1), and the absence of a variant from controls (or at an extremely low frequency if recessive) in the exome/genome database (PM2), were classified as strongly pathogenic. Genotype and phenotype correlation was established using clinical information with physical examination, laboratory test reports, segregation analysis, and previous publications.

### 2.6. Sanger Sequencing

Sanger sequencing was conducted to confirm the detected variants and to exclude the possibility of false positive. Primers were designed using Primer3Plus online software. Target DNA of 275 bp and 260 bp was amplified using Eppendorf Thermal Cycler (Merck, Darmstadt, Germany) and purified using PCR purification kits (QIAGEN, Germantown, MD, USA). Sequencing was done using the BigDye Terminator V3.1 Cycle Sequencing kit (Thermo Fisher Scientific, Waltham, MA, USA) at ABI genetic analyzers. Forward and reverse primer sequences used for c.1136C>A mutation were 5′TGGAGAGGAAGGTCCTAAGTACAG3′ and 5′AATGTGCTGCTGTGTCTCAGTTC3′, and for the c.1789delG mutation they were 5′GTTAGGGGGAATAACCGCTGG3′ and 5′CGATCCCATTGTTCCTTTTGCAC3′, respectively [27].

### 2.7. Sequence and Structure Analysis

The protein sequence was retrieved from UniProtKB–Q86YV9 and used for analysis. As there was not any previously experimentally determined structures in the RCSB’s PDB database, we predicted the HPS6 protein’s three-dimensional structure model from AlphaFold v2.0 (https://alphafold.ebi.ac.uk/entry/Q86YV9, accessed on 26 October 2021) [33]. Schrodinger’s PyMOL v2.5 (The PyMOL Molecular Graphics System, Version 1.2r3pre, Schrödinger, LLC) was used to visualize the 3D structure and specifically mutate residues using Mutagenesis Wizard. Figures were made showing the effect of nonsense and frameshift mutation using PyMOL. Functional protein interaction networks were explored using STRING version 11.0.

## 3. Results

### 3.1. Clinical Features and Pedigree Analysis

**Figure 1:**
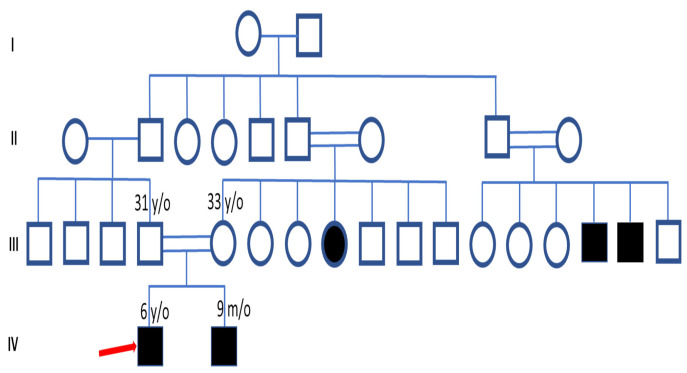
Pedigree of a family with suspected oculocutaneous albinism showing autosomal recessive mode of disease segregation: red arrow showing proband (IV-1). Brother (IV-2) has a similar problem, maternal aunt (III-8) completely lacks pigment in body and eye, and two second-degree uncles (III-15 and III-16) also have pigmentation problems. Open squares and circles indicate males and females, respectively. Black-filled symbols showing affected members of the family. Double lines between squares and circles represent consanguineous marriage.

The proband was a 6-year-old boy with lack of eye, skin, and hair pigments, as well as nystagmus and vision problems since birth. He had a positive family history and pedigree analysis showed consanguineous marriage and that his parents were first cousins. Family history revealed that his brother (9-month-old boy) has similar symptoms of vision problems, one of his maternal aunts has a complete lack of body and eye pigment, and two second-degree uncles also have pigmentation problems (Figure 1).

### 3.2. Identification of the HPS6 Variants in OCA Patient

**Figure 2:**
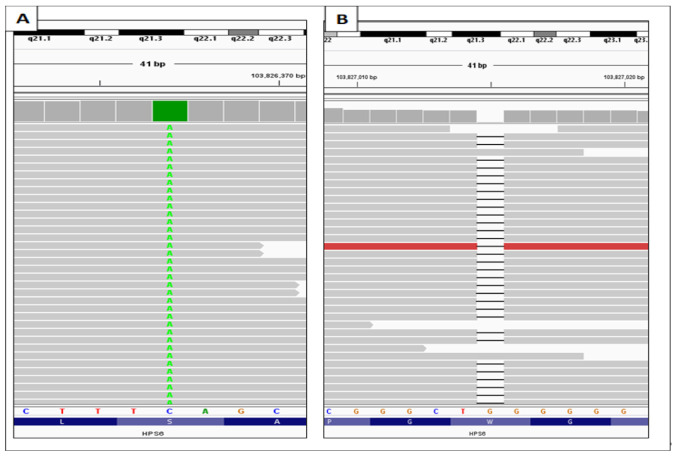
Integrative genomics viewer (IGV) visualization of the BAM files showing genetic variations. (**A**) Substitution of cytosine to adenine CTTT(C>A)AGC and (**B**) deletion of guanine [CGGGCT(delG)GGGGG].

In this study, we present the whole-exome sequencing results of OCA patients from Saudi Arabia. The parents had a consanguineous marriage (first cousin). Family pedigree indicated an AR mode of inheritance, as the two affected male children were from the same healthy parents. After applying all the filtration steps, we found two novel *HPS6* variants in the index case: a homozygous nonsense mutation (c.1136C>A, p.S379Ter) and homozygous frameshift variants (c.1789delG, p. A597GfsTer16) in exon1 of *HSP6* gene (OMIM# 607522; NM_024747.5). CADD (combined annotation dependent depletion) scores of 36 for c.1136C>A and 34 for c.1789delG strongly predict the pathogenicity. The variant (c.1136C>A) was not reported in any of the public databases including Saudi Human Genome Project (SGHP), while the frameshift variant (c.1789delG) was reported in gnomAD (MAF 0.000029) without any clinical significance, and these two potential pathogenic mutations identified in the Saudi family have not been previously described. Evaluation of BAM file confirmed the unique presence of double pathogenic homozygous variants in exon 1 of same *HPS6* gene with an altered allele read depth of 212 (c.1136C>A) and 68 (c.1789delG) with a 100% variant read frequency (Figure 2). Homozygous mutations in *HSP6* genes are known to cause Hermansky-Pudlak syndrome 6 (OMIM# 614075).

### 3.3. Validation of HPS6 Mutations

**Figure 3:**
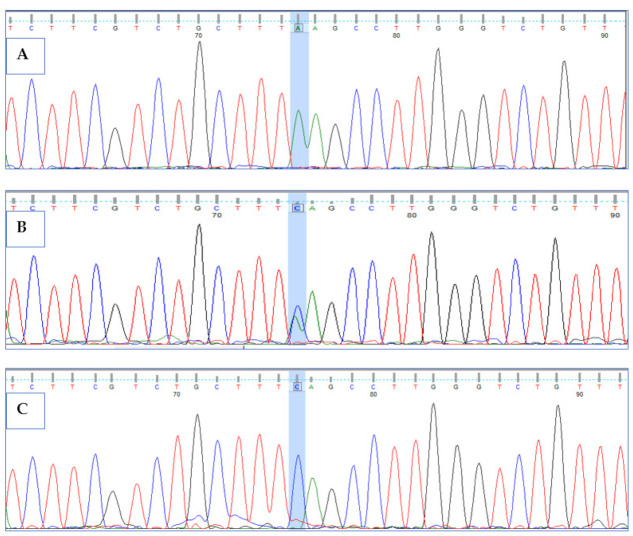
Sanger sequencing chromatograms showing the homozygous missense variant (c.1136C>A) with flanking sequence of CTTT[C/A]AGCC in exon1 of the *HPS6* gene: (**A**) homozygous altered allele (variant) in proband IV-1, (**B**) heterozygous allele in unaffected father and mother, and (**C**) homozygous wild-type allele in healthy control. Shaded chromatogram is highlighting the site of mutation.

**Figure 4:**
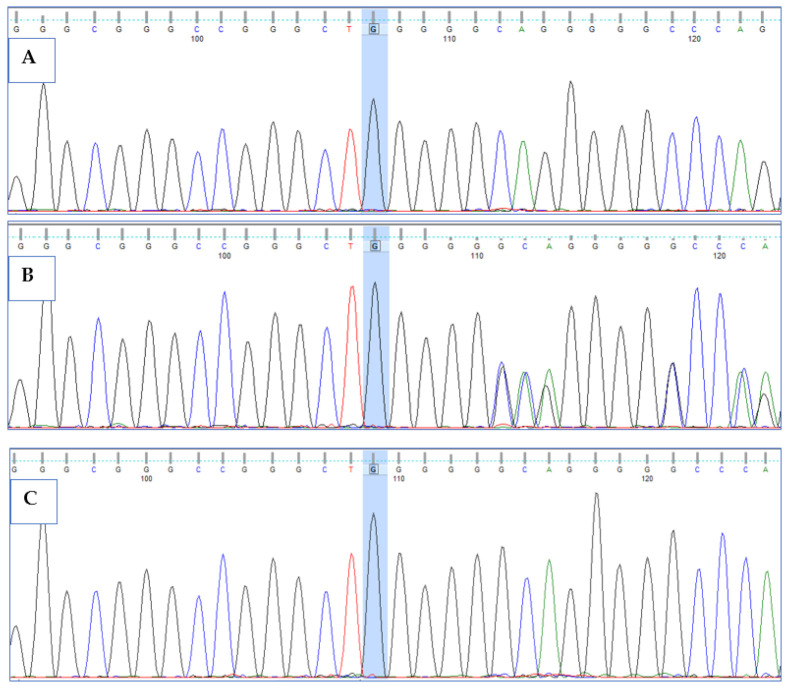
Sanger sequencing chromatograms showing deletion of G (c.1789delG) with flanking sequence CCGGGCT[G/-]GGGGGCAG in exon1 of *HPS6* gene: (**A**) homozygous deletion of G in proband, (**B**) heterozygous deletion of G in unaffected father and mother, and (**C**) homozygous wild-type allele in healthy population. Shaded chromatogram is highlighting the site of mutation.

Primers were designed for the two variants detected by WES in proband and were used to validate the mutations by Sanger sequencing analysis in proband and other available family members. Both the mutations (Ser379Ter*; Ala597GlnTer16*) were confirmed in the patient and parents through electropherograms. Double homozygous mutations in *HPS6* gene led to *HPS6* syndrome, resulting in oculocutaneous albinism in the patient, while the parents were heterozygous carriers for both variants. To rule out the possibility of pathogenic variant in healthy population, we confirmed the wild-type allele by Sanger sequencing in 10 unrelated control individuals (Figure 3 and Figure 4).

### 3.4. Computational Analysis of HPS6 Mutations

**Figure 5:**
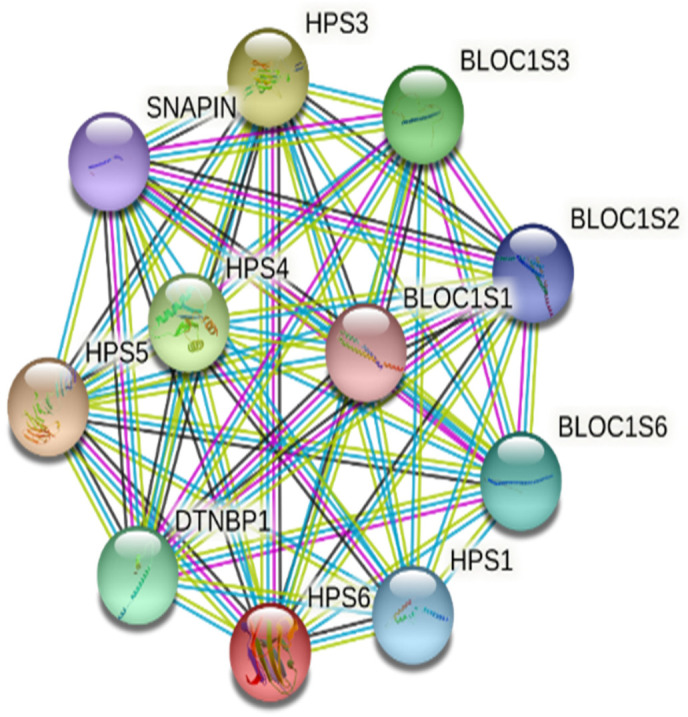
Protein–protein interaction network for *HPS6*, predicted by STRING.

**Figure 6:**
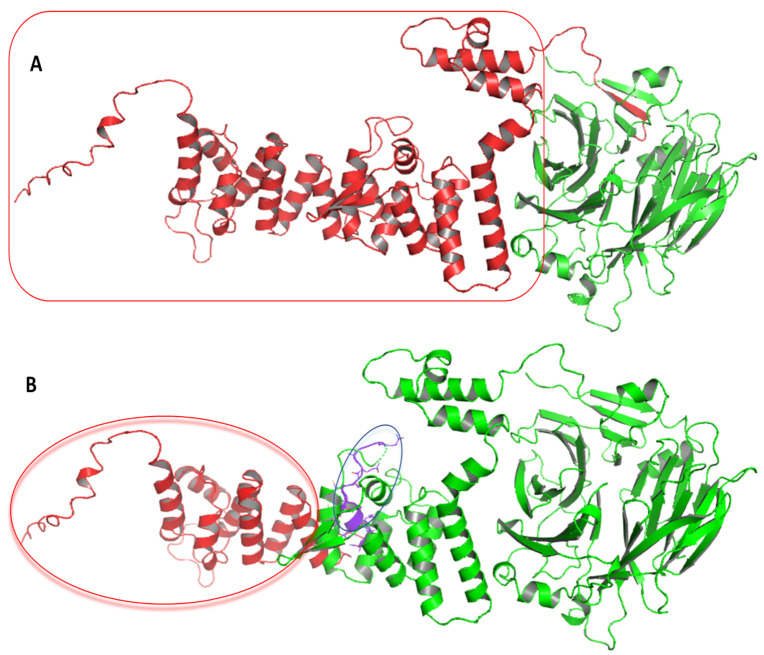
*HPS6* structure showing effect of mutations. (**A**) Red color showing truncated domain of HPS6 protein (379–775 amino acids) resulted from a nonsense mutation, and green colors represent the translated region (1–378 aa), (**B**) Purple, red, and green colors show the substituted region (597–612 aa), truncated region (613–775 aa), and translated region (1–612 aa) of the HPS6 protein models, respectively.

HPS6 protein interacts with other network proteins, such as HPS1, HPS3, HPS4, HPS5, BLOC1S1, BLOC1S2, BLOC1S3, BLOC1S6, and SNAPIN, to regulate the melanin biosynthesis pathway [34] (Figure 5). The impact of the mutations at the protein structure was determined by modeling (AlphaFold) and 3D structure visualization (Schrodinger’s PyMOL). For the first nonsense mutation, the substitution of cytosine to adenine converts serine (TCA) to termination codon (Ochre) (TAA) at p.379, resulting in a half-truncated protein. For the second frameshift mutation, the sequence analysis showed the substitution of WGAGGPGLPLYRRALAVLG by WGQGAQDCPCIAELWQC Ter* (p.597−612) followed by the truncation of the N-terminal domain (p.613-775). As both mutations are present on the same gene in a homozygous condition, the transcript will have both mutations, but the translated protein will terminate early (p.379) because of first nonsense mutation, and no further translation will happen in reality. However, two *HPS6* prediction models were developed to evaluate the impact of both mutations (Figure 6). The prediction model of wild-type *HPS6* protein with 775 amino acids had a C-score of −1.55, an estimated TM score of 0.52 ± 0.15, and an estimated RMSD of 12.1 ± 4.4 Å. The truncated protein model with 611 residues had a C-score of −2.82, an estimated TM-score of 0.39 ± 0.13, and an estimated RMSD of 14.8 ± 3.6 Å.

## 4. Discussion

Oculocutaneous albinism is a congenital pigmentation disorder of genetically heterogeneous nature. Mutations in *TYR, TYRP1, TYRP2, OCA2, OCA5, SLC45A2 SLC24A5*, and *C10orf11* are associated with eight different types of OCA [8,10,12]. Additionally, Hermansky-Pudlak syndrome (HPS), Chediak–Higashi syndrome (CHS), and X-linked ocular albinism are also associated with OCA [12,21,22,35]. Whole-exome sequencing provides an efficient approach to detect causative mutations in coding genes at higher coverage (100×) as it reduces the probability of false positive or negative results [36,37,38,39]. In this study, we identified two novel deleterious mutations in the *HPS6* gene in the *HPS6* and/or OCA family from Saudi Arabia.

Homozygous or compound heterozygous mutations in *HPS6* gene are known to cause *HPS6* (MIM #614075). To date, 25 pathogenic *HPS6* mutations, including 2 from Saudi Arabia (c.1644delA, p.Gly550Glufs*2 and c.288G>A, p.Trp96*), have been registered in the Human Gene Mutation Database [12,17,21,22,35,37,40,41,42] (Supplementary Table S1). However, the two potential pathogenic mutations identified in affected family in the present study have not been previously described.

Hermansky-Pudlak syndrome (HPS) genes products are involved in the formation of four distinct protein complexes that participate in the formation and trafficking of a group of cell structures called lysosome-related organelles (LROs). Normal LROs have been identified in pigment-producing melanocyte cells, blood-clotting platelets, and lung cells. Still, many of the molecular and cellular mechanisms underlying HPS remain unknown. Mutations prevent the formation of LROs or impair their functioning to cause Hermansky-Pudlak syndrome, a genetically heterogeneous autosomal recessive disorder characterized by oculocutaneous albinism, which may or may not be associated with frequent nosebleeds. The nine types of HPSs are distinguished by their distinct signs and symptoms and underlying genetic cause. Types 1 and 4 are associated with the most severe forms, and types 3, 5, or 6 have the mildest symptoms of the disorder, while not much is known about the signs, symptoms, and severity of types 7, 8, and 9. The *HPS6* gene mutation causes *HPS6*, which is characterized by oculocutaneous albinism, a mild bleeding diathesis, and milder pulmonary fibrosis. *HPS6* affects many organs including the eyes, nose, teeth, nasopharynx, skin, nails, hair, central nervous system, and blood. However, photophobia, prolonged bleeding on dental extractions, slow nail growth, and global developmental delay were reported in one patient only. This clearly indicates the incomplete penetrance of the *HPS6* mutation and variable phenotypes in individuals.

A limited number of *HPS6* cases with variable phenotypes makes it hard to establish a perfect genotype–phenotype correlation [17,43,44]. Miyamichi et al. (2016) reported two Japanese sisters, 4-year-old and 6-month-old girls with novel compound heterozygous mutations in *HPS6* (c.1898delC and c.2038C>T), with OCA including light brown hair and fair skin, and congenital nystagmus but no platelet dysfunction and no bleeding manifestations [17]. Schreyer-Shafir et al. (2006) reported multiple cases within a consanguineous Israeli Bedouin family with a novel insertion mutation *HPS6* (c.1066-1067insG) and HPS phenotype characterized by OCA with minimal bleeding tendency [43]. Huizing et al. (2009) reported four cases of *HPS6* including a 36-year-old woman with a novel two-base deletion (c.1865_1866delTG) and suffering with a two-vessel umbilical cord, an imperforate anus, several urinary tract infections, nystagmus, and partial albinism until 26 years, when the bleeding complications appeared. Another 13-year-old girl *HPS6* patient with compound heterozygous for two nonsense mutations (c.223C>T and c.1234C>T) had horizontal nystagmus and oculocutaneous albinism without any major bleeding problem in early childhood [44]. In present study, the patient did not have any bleeding issue but other features like absence of pigment on the skin, hair, and eyes, as well as nystagmus and vision problems, were fair to suspect with OCA. Incomplete penetrance and variable onset of disease could be the possible reason for the missing prolonged bleeding problem as it was found in couple of cases that the problem starts during adulthood. However, our diagnosis suggests that *HPS6* and the phenotypic definition of HPS need to be broadened.

Mutations in the *HPS6* gene are known to cause reduced or diminished pigmentation (albinism) in the eyes, skin, and hair. The impact of the detected *HPS6* variants (c. 1136C>A, p.Ser379Ter, c.1789delG, Ala597GlnfnTer16) were evaluated at the transcript level, and classified as likely pathogenic according to ACMGG guidelines [32]. The nonsense mutation causes premature termination of protein, and truncated proteins are usually not expressed since nonsense mRNAs are most often degraded. However, the *HPS6* transcript might escape degradation occasionally and the translated truncated protein results in a non-functional protein that dysregulates the molecular pathways by abnormal interaction with network proteins. The early termination, because of nonsense and frameshift mutations, increases the pathogenicity and affects the biogenesis of lysosome-related organelles complex-2 (BLOC2) complex formation [45,46].

Translated protein will be truncated at 379aa and no actual reflection of the frameshift mutation could be seen, as both mutations are present in the same gene, and the nonsense mutation (p.Ser379Ter) occurred before the frameshift mutation (Ala597GlnfnTer16). Disturbance in *HPS6* along with interacting partners hinders the endosomal protein-trafficking machinery and other membrane-trafficking pathways downstream [34,44,47].

Double mutation is a rare probability when two mutations are present on one allele and inherited from a single parent. We reported two different mutations carried by the same allele of *HPS6* gene where one of the double mutations was a nonsense mutation and another was a missense mutation (Ser379Ter and Ala597GlnfsTer16). Both parents were heterozygous for the double mutation and passed it to the patient, who was homozygous for the double mutation. Although the nonsense mutation was considered to be the main defect because of its position and impact, the frameshift substitutions induced disease-causing mutations as well. Double mutations have been described for genetic disorders, including hypertrophic cardiomyopathy, cystic fibrosis, Gaucher disease, mucopolysaccharidosis Type IVA, and aspartylglucosaminuria [48,49,50,51,52,53,54]. Studies have reported multiple double mutations (one pathogenic and another likely pathogenic) in MYBPC3 (Asp745Gly and Pro873His; Arg326Gln and Gln1233Ter; Glu1265Val and Cys1266Arg), TNNT2 (Phe110Ile and Arg130Cys), MYH7 (Arg845Gly and Thr1929Met), etc. [48,50]. These studies do not report the combined effect with double mutations, but the possible effect of two mutation acting simultaneously could be more severe than the effect caused by either mutation separately. Our findings suggest that double mutant alleles may have important implications for molecular diagnosis and genetic counselling.

## 5. Conclusions

In conclusion, two novel homozygous *HPS6* variants (c. 1136C>A, p.Ser379Ter and c.1789delG, Ala597GlnfnTer16) were detected in an OCA patient, and to the best of our knowledge, this is the first report of double *HPS6* mutations in the Saudi population. In silico structural analyses showed HPS6 protein models with significant domain loss that suggest functional impact and might dysregulate molecular pathways. The clinical features of the patient with pigmentation problems in hair and eyes suggest OCA, while identification of the *HPS6* mutation suggests that the patient may have two independent inherited disorders, OCA and *HPS6*. Thus, WES could be fairly used for diagnosis of *HPS6* and/or OCA, particularly in a consanguineous family.

## Data Availability

Data are available upon request to corresponding author Sajjad Karim, skarim1@kau.edu.sa. The dataset for this article is not publicly available because the family did not consent to share data publicly.

## Supplementary Materials

The following are available online at https://www.mdpi.com/article/10.3390/life12010014/s1, Table S1: List of clinically significant variants of *HPS6* gene.

## Abbreviations

OCA	Oculocutaneous albinism
HPS	Hermansky-Pudlak syndrome
WES	Whole-exome sequencing
ACMGG	American College of Medical Genetics and Genomics
HGVS	Human Genome Variation Society
CADD	Combined Annotation-Dependent Depletion
