# Supplementary material for: Two Novel Homozygous HPS6 Mutations (Double Mutant) Identified by Whole-Exome Sequencing in a Saudi Consanguineous Family Suspected for Oculocutaneous Albinism
Source: Life (Basel). 2021 Dec 23;12(1):14. doi: 10.3390/life12010014 (PMC8779141; doi:10.3390/life12010014)
Supplement: Supplementary file 1 [file life-12-00014-s001.zip › life-1475969-supplementary.pdf]

Supplementary Table 1: List of clinically significant variants of HPS6 gene

S.No	*SNP ID*	*Clinical Significance*	*Variation*	*Type*
1	*rs1590262450*	*Pathogenic*	*NM_024747.5(HPS6):c.155delT (p.Val52fs)*	*Frameshift*
2	*rs1590263807*	*Pathogenic*	*NM_024747.5(HPS6):c.1624delG (p.Asp542fs)*	*Frameshift*
3	*rs1131692332*	*Pathogenic*	*NM_024747.5(HPS6):c.1898delC (p.Pro633fs)*	*Frameshift*
4	*rs1131692333*	*Pathogenic*	*NM_024747.5(HPS6):c.2038C>T (p.Gln680Ter)*	*Nonsense*
5	*rs1220869113*	*Pathogenic*	*NM_024747.5(HPS6):c.1711_1712insAG (p.Cys571Ter)*	*Nonsense*
6	*rs1564899492*	*Pathogenic*	*NM_024747.5(HPS6):c.1065dupG (p.Leu356fs)*	*Frameshift*
7	*rs281865107*	*Pathogenic*	*NM_024747.5(HPS6):c.223C>T (p.Gln75Ter)*	*Nonsense*
8	*rs281865109*	*Pathogenic*	*NM_024747.5(HPS6):c.815C>T (p.Thr272Ile)*	*Missense*
9	*rs281865110*	*Pathogenic*	*NM_024747.5(HPS6):c.913C>T (p.Gln305Ter)*	*Nonsense*
10	*rs281865112*	*Pathogenic*	*NM_024747.5(HPS6):c.1234C>T (p.Gln412Ter)*	*Nonsense*
11	*rs281865113*	*Pathogenic*	*NM_024747.5(HPS6):c.1714_1717delTGTC (p.Leu572fs)*	*Frameshift*
12	*rs281865114*	*Pathogenic*	*NM_024747.5(HPS6):c.1865_1866delTG (p.Leu622fs)*	*Frameshift*
13	*rs1564899012*	*Pathogenic*	*NM_024747.5(HPS6):c.283delG (p.Val95fs)*	*Frameshift*
14	*rs1564899951*	*Pathogenic*	*NM_024747.5(HPS6):c.1864_1871delGCTCTGGA (p.Leu622fs)*	*Frameshift*
15	*rs1478574193*	*Likely Pathogenic*	*NM_024747.5(HPS6):c.779G>A (p.Gly260Glu)*	*Missense*
16	*rs1590263820*	*Likely Pathogenic*	*NM_024747.5(HPS6):c.1649delG (p.Gly550fs)*	*Frameshift*
17	*rs756325364*	*Likely Pathogenic*	*NM_024747.6(HPS6):c.823C>T (p.Pro275Ser)*	*Missense*
18	*rs756471925*	*Likely Pathogenic*	*NM_024747.6(HPS6):c.706_707delTC(p.Ser236fs)*	*Frameshift*
19	*1050563*	*Likely Pathogenic*	*NM_024747.6(HPS6):c.206_210dupCGGGC (p.Trp71fs)*	*Frameshift*
20	*996365*	*Likely Pathogenic*	*NM_024747.6(HPS6):c.1999C>T (p.Arg667Ter)*	*Nonsense*
21	*996366*	*Likely Pathogenic*	*NM_024747.6(HPS6):c.335G>A (p.Trp112Ter)*	*Nonsense*
22	*996367*	*Likely Pathogenic*	*NM_024747.6(HPS6):c.1732C>T (p.Arg578Ter)*	*Nonsense*
23	*rs1554903728*	*Likely Pathogenic*	*NM_024747.5(HPS6):c.1693T>G (p.Phe565Val)*	*Missense*
24	*rs1590262288*	*Likely Pathogenic*	*NM_024747.6(HPS6):c.19_20delCT (p.Leu7fs)*	*Frameshift*
25	*rs763073715*	*Likely Pathogenic*	*NM_024747.6(HPS6):c.1A>G (p.Met1Val)*	*Missense*
